# Coexistence of a novel *SV2B-ALK*, *EML4-ALK* double-fusion in a lung poorly differentiated adenocarcinoma patient and response to alectinib: a case report and literature review

**DOI:** 10.3389/fonc.2024.1453259

**Published:** 2024-12-13

**Authors:** Huang Chen, Menglan Zhang, Liyan Bai, Yun Niu, Xiaowei Wang, Ruiying Jiang, Ye Wang, Qianqian Feng, Bei Wang, Tingli Dai, Mingming Yuan, Rongrong Chen, Yujuan Qi, Dingrong Zhong

**Affiliations:** ^1^ Department of Pathology, China-Janpan Friendship Hospital, Beijing, China; ^2^ Department of Pathology, Qinghai Provincial People’s Hospital, Xining, China; ^3^ Department of Oncology, Qinghai Provincial People’s Hospital, Xining, China; ^4^ Geneplus-Beijing, Beijing, China

**Keywords:** alectinib, *ALK* double fusions, CNS metastases, NSCLC, *SV2B-ALK* novel fusion

## Abstract

**Background:**

Anaplastic lymphoma kinase (*ALK*) rearrangement, the most common oncogenic rearrangement in lung adenocarcinoma, occurs in approximately 5% of non-small cell lung cancer (NSCLC) patients. *EML4* gene is the most common partner of *ALK* rearrangement, and distinct EML4-ALK fusions differ in their responsiveness to ALK tyrosine kinase inhibitors. However, the concurrence of two *ALK* rearrangements in one patient and whose response to ALK-TKIs have rarely been reported so far.

**Case presentation:**

A 47-year-old Chinese male was diagnosed with stage IV lung adenocarcinoma with multiple intracranial metastases and adrenal metastasis. After progression of two lines of chemotherapy combined with local radiotherapy regimens, his tumor tissue sample was sent to perform the DNA-based next-generation sequencing of 116 genes. Surprisingly, *EML4-ALK* (E13:A20) fusion and a novel *SV2B-ALK* (S6:A20) fusion were concurrently identified, which was confirmed using immunohistochemistry and fluorescence *in-situ* hybridization. Given the superior efficacy of alectinib, the patient received alectinib in the third-line setting with the progression-free survival over 14 months up to now. Moreover, through comprehensive review of previous literatures, a total of 22 patients with multiple *ALK* fusions and their response to ALK-TKIs were summarized.

**Conclusion:**

This is the first report of a NSCLC patient with a novel *SV2B-ALK*, *EML4-ALK* double-fusion benefiting from alectinib. Alectinib may be an effective therapeutic option for both primary and metastatic lesions including brain metastases in the late-line setting in NSCLC patients with double-*ALK* fusion.

## 1 Introduction

Lung cancer is the leading cause of cancer-related mortality, among which non-small cell lung cancer (NSCLC) is the most predominant type (1). *ALK* rearrangement was found in 3% to 7% of NSCLC patients in previous studies and *EML4* gene is the most common *ALK* rearrangement partner (2). Multiple ALK tyrosine kinase inhibitors (TKIs) have been proven to greatly improve the clinical outcome of *ALK*-rearranged NSCLC patients (3, 4). Currently, more and more *ALK* fusions have been reported with the wide application of comprehensive next-generation sequencing (NGS) (2, 5). However, reports on patients harboring double *ALK* fusions simultaneously were still rare, and the effectiveness of ALK-TKIs in these patients was also barely reported. Herein, we presented one NSCLC patient with extensive metastases and a novel synaptic vesicle protein 2B (*SV2B*) - *ALK* and *EML4 - ALK* double-fusion. This patient responded well to alectinib in the third-line setting with the progression-free survival (PFS) exceeding 14 months up to now. Moreover, the previous reports of *ALK* double fusions were summarized to facilitate clinicians to acquire the clinical evidences and make clinical decisions for these even rare patients with *ALK* double fusions.

## 2 Case presentation

**Figure 1:**
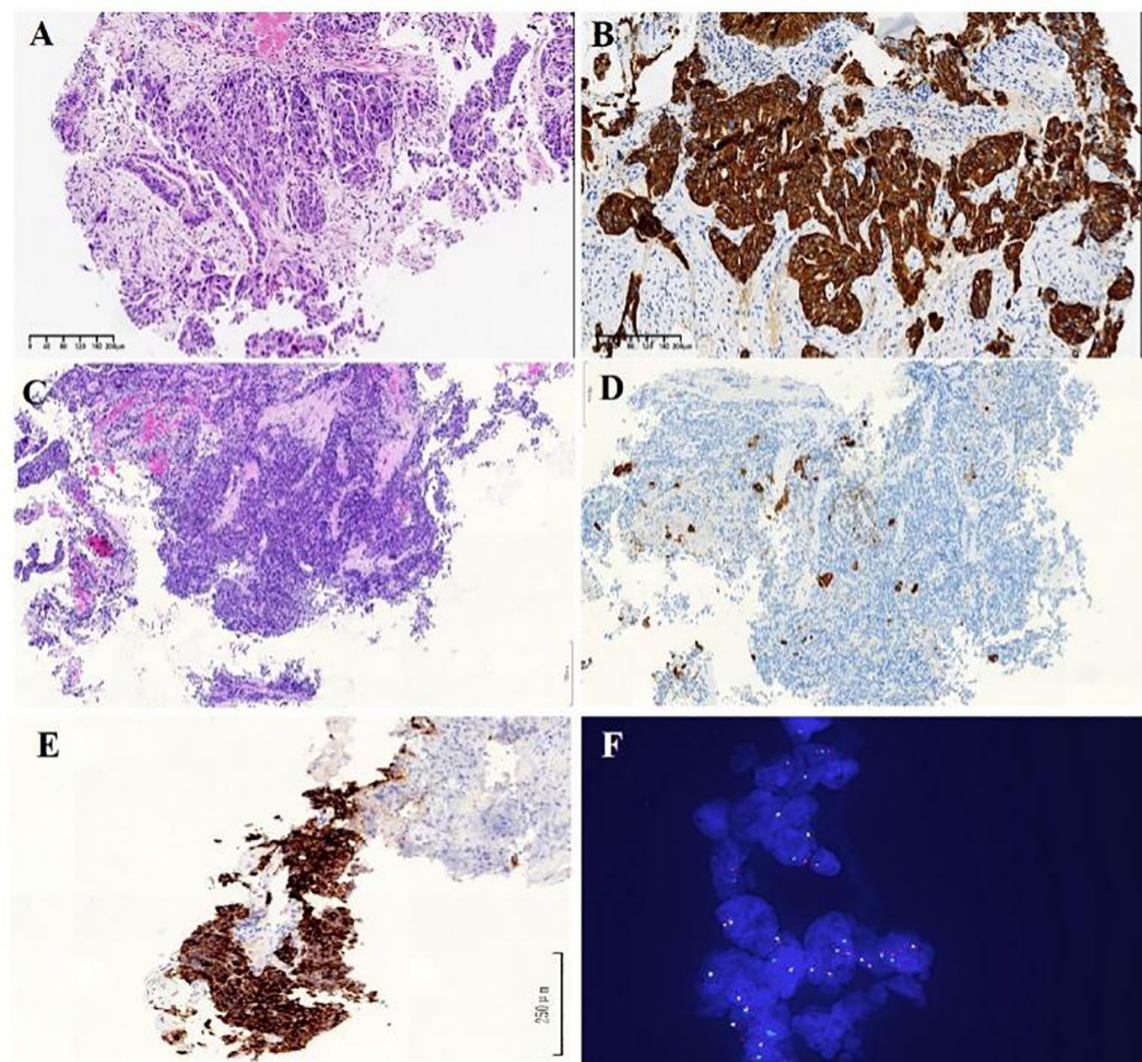
**(A)** HE staining of transbronchial biopsy under fiberscopic examination indicates poorly differentiated NSCLC Diagnosis of lung adenocarcinoma. **(B)** Immunohistochemistry analysis revealed immunoreactivity to CK7 (×100). **(C)** HE staining of re-biopsy of the lesion show as solid-pattern adenocarcinoma. **(D)** Immunohistochemistry analysis revealed immunoreactivity to CK7 (×100). **(E)** Immunohistochemistry staining showed strong ALK receptor tyrosine kinase protein expression in the re-biopsy tissue (×200). **(F)** Fluorescent *in situ* hybridization showed rearranged *ALK* gene through *ALK* gene isolation probe (×100).

A 47-year-old Chinese male with the smoking history of 30 pack-year came to Qinghai Provincial People’s Hospital complaining of coughing and expectoration. Chest computed tomography (CT) showed 4.0×3.3 cm mass in the lower lobe of the right lung with metastasis to hilum of right lung, mediastinal lymph node and supraclavicular lymph node. Magnetic resonance imaging (MRI) scans indicated multiple intracranial metastases and adrenal metastasis. Transbronchial biopsy under fiberscope revealed poorly differentiated adenocarcinoma (cT3N2M1c, stage IVb, **Figures 1A, B**). EGFR wild type was identified in the biopsy tumor tissue through DNA-based PCR. The mutations of other driver genes, including *KRAS*, *ALK* and *ROS1*, were not detected. This patient received chemotherapy (cisplatin/pemetrexed/bevacizumab) for 8 cycles and radiotherapy were added in lung metastases (60Gy/2Gy/30F), intracranial metastases (3000cGy/10Fx) and adrenal metastasis (6000cGy/30Fx). Progression occurred after 18 months of treatment and albumin-bound paclitaxel was administered as second-line chemotherapy. After 1 cycle of chemotherapy, the patient’s symptoms of chest tightness and shortness of breath were aggravated, and CT scan revealed enlargement of the lesions in right lung and bilateral adrenal metastases.

**Figure 2:**
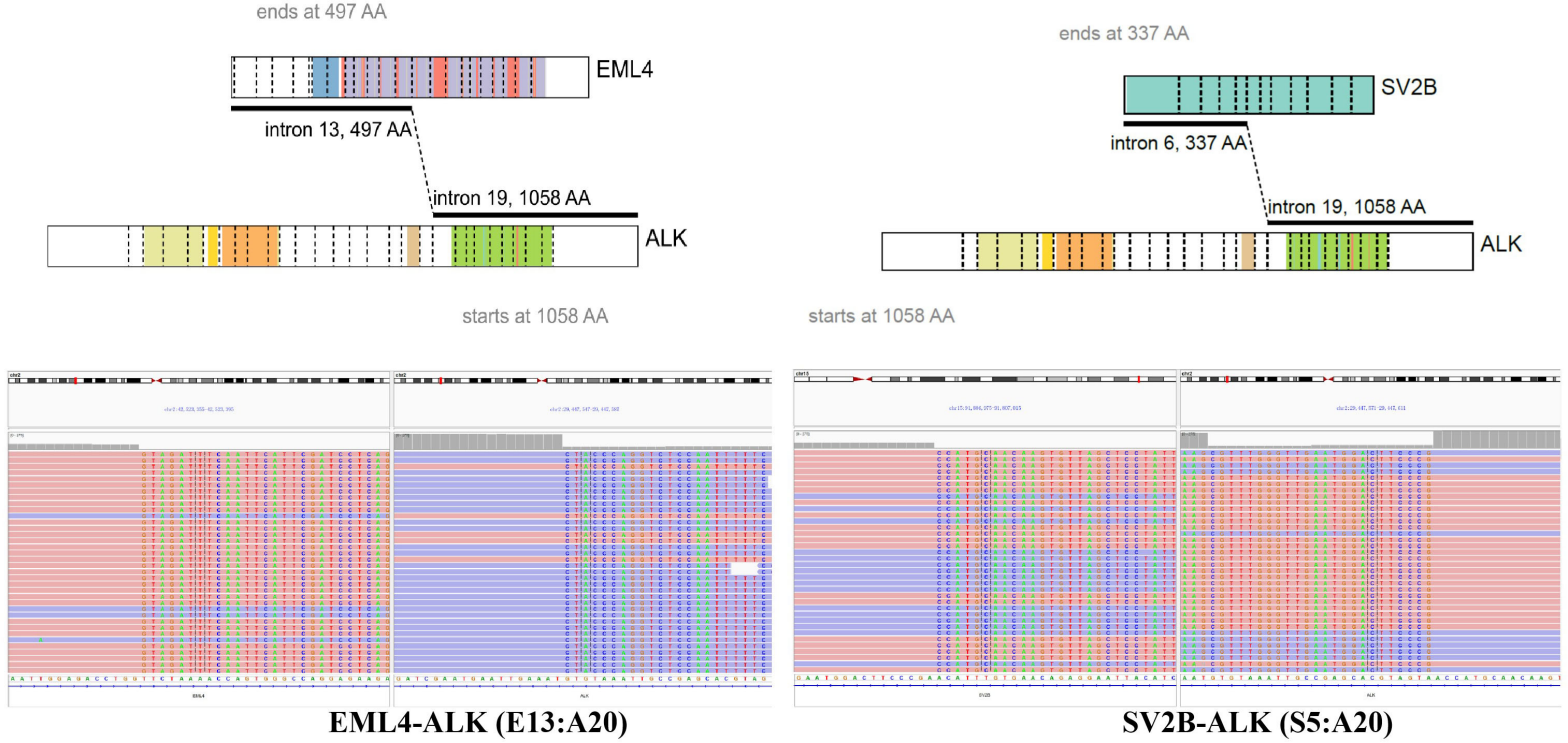
Identification of *SV2B-ALK* and *EML4-ALK* double-fusion. ALK, anaplastic lymphoma kinase; SV2B, neurobeachin; EML4, echinoderm microtubule-associated protein-like 4 gene.

A re-biopsy of the growing lung lesion was performed and pathological analysis confirmed it as solid-pattern adenocarcinoma. Immunohistochemistry analysis was positive for thyroid transcription factor 1, NapsinA, and 30% for Ki-67, but negative for chromograin A, CD56, and synaptophysin (**Figures 1C, D**). The tissue sample was also sent to perform the DNA-based NGS (Amoy Diagnostics, Xiamen, China) using a gene panel comprising of 116 lung cancer-related genes. Two *ALK* rearrangements, including *EML4-ALK* (E13:A20) and a novel *SV2B-ALK* (S6:A20), were concurrently identified with the abundance of 43.81% and 41.01%, respectively (**Figure 2**). Besides, a synonymous mutation in exon 4 of *TP53* (c.375G>A, allelic frequency: 27.32%) and a nonsense mutation in exon 2 of *CDKN2A* (c.358G>T, allelic frequency: 20.64%) were also detected. To confirm the presence of *ALK* fusion, the expression of ALK protein was evaluated using a rabbit monoclonal antibody (Ventana D5F3, ROCHE, China) on a benchmark system (**Figure 1E**), which reveals strong expression of ALK protein in the lung lesion. Fluorescent *in situ* hybridization (FISH) through *ALK* gene isolation probe also confirmed the ALK rearrangement (**Figure 1F**).

**Figure 3:**
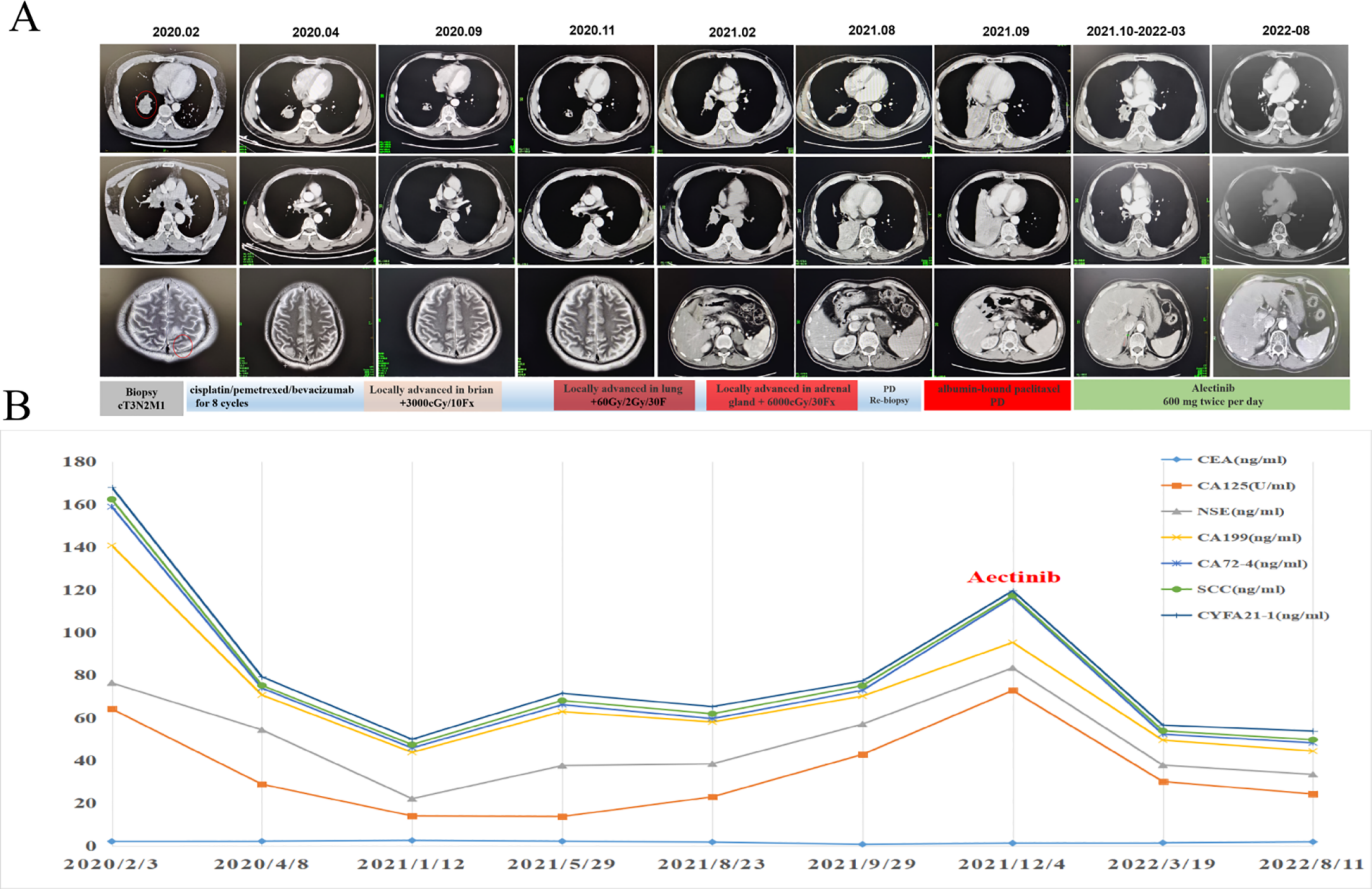
Dynamic changes of plasma tumor markers and tumor lesions during the alectinib treatment. **(A)** CT/MRI scans before and during alectinib treatment. **(B)** Dynamic monitoring of tumor markers.

Given the promising efficacy regarding both central nervous system (CNS) and non-CNS lesions and tolerability of alectinib, alectinib was administered orally at a dose of 600 mg twice per day as the third-line treatment from October 2021 to December 2022. Dynamic monitoring of serum tumor markers suggested that CEA, CA125 and CA19-9 dropped dramatically five month later (**Figure 3A**). A follow-up CT scan performed at 10 months after treatment found that the lesions in right lung obviously shrank from 3.3 cm * 3.0 cm to 0.9 cm * 1.0 cm, and the right adrenal metastasis was also smaller than before (4.9 cm *3.0 cm to 4.1 cm * 2.0 cm). Meanwhile, MRI examination showed the intracranial metastases in bilateral frontal lobe and parietal lobe were not clearly displayed, and the left occipital lobe lesions were smaller than before (**Figure 3B**). According to RECIST 1.1, partial response was achieved. The patient’s chest tightness, and shortness of breath were significantly relieved. The patient tolerated the treatment well with no significant adverse events until May 2023, when the patient succumbed to respiratory complications secondary to COVID-19 infection.

## 3 Discussion

*EML4-ALK* rearrangement defines a unique molecular subtype of NSCLC, of which the patients could benefit from multiple ALK inhibitors. With the wide application of extensive genomic sequencing, more than 50 fusion partners of *ALK* have been found in NSCLC (6). It is worthwhile to report the uncommon *ALK* partners and their sensitiveness to ALK inhibitors, which may provide the clinical evidences of treatment options to patients with the same rare fusion. To our knowledge, this is the first study to report a lung adenocarcinoma patient with a novel *SV2B-ALK* and *EML4-ALK* double fusion who had a durable and remarkable response to alectinib in the third-line setting.

Alectinib is a second-generation, highly-selective ALK inhibitor and is highly recommended by NSCLC NCCN guideline due to its excellent efficacy in *ALK*-rearranged NSCLC patients. In the first-line setting, PFS was significantly prolonged with alectinib vs. crizotinib (median PFS: 34.8 months vs. 10.9 months) (7). In Asian patients, the median PFS of alectinib and crizotinib was 41.6 months and 11.1 months, respectively. Patients with CNS metastases at baseline also respond well to alectinib with the median PFS of 42.3 months (8). In ALEX-J study, the median PFS of alectinib was 20.3 months in ALK inhibitor-naive, chemotherapy-treated NSCLC patients (9). The efficacy of alectinib in third-line setting has not been well studied. In our case, the patient received alectinib as the third-line therapy after treatment failure of chemotherapy and radiotherapy with the excellent efficacy in both non-CNS and CNS lesions, which suggested that alectinib could be considered in the third-line setting in patients with CNS metastases.

In our case, 1-13 exons of *EML4* gene fused with 20-29 exons of *ALK*, which was a classic *EML4-ALK* v1 fusion. Moreover, 1-6 exons of *SV2B* gene fused with 20-29 exons of *ALK* gene, which retained the intact kinase domain of ALK protein. Synaptic vesicle protein (SV2) is a neuronal protein with three isoforms (SV2A, SV2B and SV2C), and plays an important role in exocytosis and in the secretory process of synaptic and endocrine cells. As a synaptic protein, SV2B is widely expressed in the nervous system, especially throughout the brain (10, 11). Thus, we speculated that the promoter of *SV2B* driven *ALK* kinase domain expression may contribute to brain metastasis of this patients, as well as to the excellent intracranial efficacy of alectinib, though we could not confirm its expression in the brain lesion. With similar dilemma of cases reporting multiple ALK fusions, we summarized previous literatures to facilitate future decision making. Up to now, only 22 cases have been reported (**Table 1**) (6, 12–31). Among them, 12 patients harbored the classic *EML4-ALK* fusion and an uncommon *ALK* fusion (6, 12, 13, 15, 18, 20, 22, 25, 27–29, 31). Other patients had two or more uncommon *ALK* fusions. *STRN-ALK* fusion was reported in two cases (14, 15), while other uncommon fusions occurred only once. In several studies, these fusions were confirmed using other techniques, including FISH, IHC, Sanger sequencing and PCR. Most patients were confirmed to be positive for *ALK* rearrangement or expression, whilst P022 had negative result for FISH testing (31). In previous reports, most patients received crizotinib as the systematic therapy with the longest PFS over 31 months (13). In the past two years, alectinib was also used in 5 cases with the immature PFS data in 4 of them. It’s worth noting that P21 received crizotinib as second-line therapy. CT scans after 3 months of treatment showed a significant peripheral response, but growing brain metastases (30). Considering the superior efficacy of aletinib over crizotinib and its efficacy against brain metastases, alectinib was selected as the third-line therapy in this case, with the PFS over 14 months and prominent response in brain metastases.

**Table 1:** Literature review of the cases with ALK multiple fusions.

PMID	ID	Year	Age	Sex	Smoking Status	Cancer Type	Clinical stage	ALK fusions					Co-mutations	ALK-TKIs Treatment			Reference
								NGS sequencing	FISH	IHC	PCR	Sanger sequencing		Line	ALK TKI	PFS	
35144623	P01	2022	38	F	NS	ADC	T2N0M1b	EML4(PMT..EX6)-ALK(EX20..END) (DNA+RNA) SSH2(END..EX3)-ALK (EX19..END) (DNA+RNA)	NA	+	NA	NA	NA	3	Crizotinib	8m(severe AEs)	(12)
35144623	P02	2022	58	F	NS	ADC	T4N2M1b	EML4(PMT..EX20)-ALK(EX20..END)(DNA+RNA) ARID2(PMT..EX12)-ALK(EX23..END)(DNA+RNA)	NA	+	NA	NA	NA	1	Crizotinib	12m	(12)
34763158	P03	2021	39	F	NS	ADC	T4N3M1c	EML4(PMT..EX13)-ALK(EX20..END) NBEA(PMT..EX5)-ALK(EX20..END)	+	NA	NA	NA	SETD2 c.1320 T> A	2	Alectinib	11+m	(6)
34754197	P04	2021	55	F	NA	ADC	T2aNxM1b	EML4-ALK(E6:A20) TAC1-ALK(Intergenic:A20)	NA	NA	NA	NA	ALK p.F1174C	1	Crizotinib+Radiotherapy	14m	(13)
														2	Crizotinib+Bevacizumab	6m	(13)
														3	Salvage surgery+Crizotinib	31+m	(13)
34485156	P05	2021	29	F	NS	ADC	T2N2M1c	PDK1-ALK(P7:A20) STRN-ALK(S3:A20)	NA	+	NA	NA	TP53	1	Alectinib	7+m	(14)
34232939	P06	2021	38	M	NS	ADC	TxNxM1b	EML4(PMT..EX2)-ALK(EX20..END) STRN(PMT..EX3)-ALK(EX20..END)	NA	NA	NA	NA	TP53, RB1, EGFR L858R, T790M	2	Crizotinib+Osimertinib	5m	(15)
34589958	P07	2020	80	M	NA	NEC	TxNxM1c	MRPL13(PMT..EX3)-ALK(EX20..END) PPP1CB(PMT..EX5)-ALK(EX20..END)	NA	NA	NA	NA	EGFR L858R amplification RET amplification CDKN2A copy number loss	2	Crizotinib+Osimertinib	1m (severe AEs, death)	(16)
33419583	P08	2021	51	M	NA	ADC	TxNxM1c	CDCA7-ALK(C intergenic:A19) FSIP2-ALK(F intergenic:A18) ALK-ERLEC1(A20:E intergenic)	NA	+	NA	NA	NA	1	Crizotinib	2+m	(17)
33091968	P09	2021	61	M	NS	ADC, IMT	NA	LOC101927285(intergenic)-ALK(EX20..END) (DNA)→EML4-ALK(RNA)(adenocarcinoma) TPM3(PMT..EX8)-ALK(EX20..END) (IMT) (DNA)	NA	+	NA	NA	CTNNB1 c.133T>C (ADC) GNA11 c.844A>G (IMT)	1	Crizotinib	17m	(18)
														2	Alectinib	4+m	(18)
33637344	P10	2021	43	F	NA	ADC	T4N2M1a	THUMPD2(PMT..EX6)–ALK(EX20..END) RGS18 (intergenic)-ALK(EX20..END)	NA	+	NA	NA	NA	1	Crizotinib	17+m	(19)
32409002	P11	2020	60	M	NA	ADC	T4N3M1a	EML4-ALK(E20:A20) ALK(PMT..EX19)-BIRC6(EX33..END)	NA	NA	NA	+	NA	1	Alectinib	7+m	(20)
33489809	P12	2020	32	M	S	NSCLC	T4N3M1a	CCNY(intergenic)-ALK(EX20..END) ATIC(PMT..EX7)-ALK(EX20..END)	+	+	NA	NA	NA	1	Crizotinib	6+m	(21)
33157918	P13	2020	55	F	NS	ADC	IIIB	EML4(PMT..EX6)-ALK(EX20..END) CDK15(PMT..EX10)-ALK(EX19..END)	NA	NA	NA	NA	NA	2	Crizotinib	23+m	(22)
32903930	P14	2020	53	M	NS	ADC	T4N2M1	COX7A2L–ALK(C intergenic:A20) LINC01210–ALK(L intergenic:A20) ATP13A4–ALK(A9:A19)	NA	NA	NA	NA	SLCO2A1–ALK(S intergenic:A18) ALK p.C1156Y	1	Crizotinib	12m	(23)
														2	Ceritinib	7m	(23)
31766077	P15	2020	47	F	NS	ADC	T2N2M1	LOC388942-ALK LINC00211-ALK	NA	NA	NA	NA	NTRK amplification FBXW7 amplification KEAP1 p.E493K	1	Crizotinib	16.3m	(24)
														2	Alectinib	18.8m	(24)
														3	Lorlatinib	8.7m	(24)
32212216	P16	2020	64	F	NS	ADC	T2aN0M1a	EML4(PMT..EX20)-ALK(EX20..END) NLRC4(EX6..END)-ALK(EX20..END)	NA	NA	NA	NA	NA	1	Crizotinib	10+m	(25)
31160357	P17	2019	73	M	S	ADC	T2bN1M0	SLMAP(PMT..EX12)-ALK(EX20..END) (RNA) SLMAP(PMT..EX13)-ALK(EX20..END) (RNA)	+	+	+	NA	NA	Adjuvant	Crizotinib	24+m (DFS)	(26)
31122560	P18	2019	29	M	S	ADC	T2N3M1	EML4-ALK(E18:A20) BCL11A-ALK(B2:A18)	NA	NA	NA	NA	NA	1	Crizotinib	13m	(27)
31757376	P19	2019	44	M	S	ADC	T3N3M0	EML4-ALK PRKCB-ALK	NA	NA	NA	NA	DDR1 p.L720V TP53 p.Q331∗ NF1 p.V1432F	1	Crizotinib	5m	(28)
														2	Ceritinib	2+m	(28)
30368418	P20	2018	56	M	NA	ADC	Relapsed	EML6-ALK FBXO11-ALK	NA	+	NA	NA	NA	5	Crizotinib	11+m	(29)
29472060	P21	2018	44	M	S	ADC	TxNxM1c	DYSF-ALK ITGAV-ALK	NA	+	NA	NA	ALK p.Q1146P MET p.M636V	2	Crizotinib	3m (brain progression)	(30)
28215724	P22	2017	NA	NA	NA	ADC	TxNxM1	EML4(PMT..EX13)-ALK(EX20..END) WDR43(EX4..Unknown)-ALK(EX19..PMT)	–	+	NA	NA	NA	NA	No	NA	(31)

ADC, adenocarcinoma; AE, adverse event; DFS, disease-free survival; F, female; FISH, fluorescence *in-situ* hybridization; IHC, immunohistochemistry; IMT, inflammatory myofibroblastic tumor; M: male; NA, not available; NEC, neuroendocrine carcinoma; NGS, next-generation sequencing; NS, non-smoker; NSCLC, non-small cell lung cancer; PCR, polymerase-chain reaction; PFS, progression-free survival; S, smoker; TKI, tyrosine kinase inhibitor.

+, the test result of ALK is positive; -, the test result of ALK is negative.

In conclusion, this is the first report of a novel *SV2B-ALK* and *EML4-ALK* double-fusion in a lung adenocarcinoma patient with extensive metastases. Our patient responded well to alectinib in both CNS and non-CNS lesions in the third line setting. By reviewing literature, we concluded that comprehensive NGS is crucial to detect the novel fusions in NSCLC, which may affect the sensitivity of targeted therapy and thus the decision-making of treatment regimens.

## Data Availability

The datasets presented in this study can be found in online repositories. The names of the repository/repositories and accession number(s) can be found in the article/supplementary material.
